# Comparative Analysis of Thromboxane B2 (TXB2) and Prostaglandin E2 (PGE2) Levels in the Gingival Crevicular Fluid (GCF) of Diabetic Patients With Chronic Periodontitis: An Enzyme Immunoassay Study

**DOI:** 10.7759/cureus.70929

**Published:** 2024-10-06

**Authors:** Aarti Y Mahale, Swati Agarwal, Rajat Bansal, Sheeba Khan, Purnita Goyel, Abhinav Chaubey, Seema Gupta

**Affiliations:** 1 Department of Biochemistry, Jawahar Medical Foundation's Annasaheb Chudaman Patil Memorial Medical College, Dhule, IND; 2 Department of Periodontics, Kothiwal Dental College and Research Centre, Moradabad, IND; 3 Department of Pedodontics and Preventive Dentistry, Kothiwal Dental College and Research Centre, Moradabad, IND; 4 Department of Conservative Dentistry and Endodontics, Kothiwal Dental College and Research Centre, Moradabad, IND; 5 Department of Orthodontics, Kothiwal Dental College and Research Centre, Moradabad, IND

**Keywords:** glycated hemoglobin, immunoassay techniques, periodontal pocket, periodontium, type 2 diabetes mellitus

## Abstract

Introduction: A persistent systemic hyperglycemia as observed in patients with type 2 diabetes mellitus (DM) has been associated with periodontal inflammation. Therefore, the primary objective of the present study was to assess the levels of thromboxane B2 (TXB2) and prostaglandin E2 (PGE2) in the gingival crevicular fluid (GCF) of type 2 DM and non-diabetic patients with chronic periodontitis. The secondary objectives were to correlate these levels with various parameters such as age, gender, glycated hemoglobin (HbA1c) levels, probing depth (PD), and fasting blood sugar levels and to determine the significant predictors for PGE2 and TXB2 levels.

Materials and methods: This case-control, cross-sectional study was conducted on 60 patients who were divided into three groups: group 1 (n=20) comprising type 2 DM patients with chronic periodontitis, group 2 (n=20) composed of non-diabetic patients with chronic periodontitis, and group 3 (n=20) as controls comprising of periodontally and systemically healthy individuals. HbA1c, fasting blood glucose levels, PD, and PGE2 and TXB2 levels were checked in GCF for all the patients. The data was subjected to statistical analysis.

Results: The two-way analysis of variance test results revealed statistically significant differences across groups for all parameters. The levels of both biomarkers showed a positive correlation with HbA1c, PD, fasting blood sugar levels, and duration of type 2 DM. Furthermore, PD and fasting blood sugar levels showed the strongest influence on both PGE2 and TXB2 levels. For PGE2, fasting blood sugar levels (p=0.006) and PD (p<0.001) were significant predictors. For TXB2, significant predictors included HbA1c (p=0.003), fasting blood sugar levels (p=0.015), and PD (p<0.001).

Conclusion: PGE2 and TXB2 levels were significantly increased in type 2 DM with chronic periodontitis, compared with non-diabetic patients with chronic periodontitis and periodontally healthy patients.

## Introduction

Periodontal inflammation, a prevalent oral health issue, is characterized by clinical manifestations such as bleeding on probing, bone loss, and probing depth (PD) ≥3 mm. If not accurately diagnosed and promptly treated, the persistent inflammation of the periodontal soft tissues may progress, leading to the degradation of osseous structures within the dental socket, ultimately culminating in the loss of teeth [1]. Microbial metabolites in periodontitis stimulate the secretion of inflammatory mediators, notably prostaglandin E2 (PGE2) and thromboxane B2 (TXB2), whose dysregulation within the gingival crevicular fluid (GCF) may contribute to the pathogenesis of the disease. Its multifaceted roles encompass the modulation of inflammation, immune responses, vasodilation, and nociception [2].

PGE2 induces vasodilation, enhances vascular permeability, and subsequently modulates the synthesis of osteoclast-activating factors, thereby promoting bone resorption. The concentration of PGE2, which is detectable in the GCF of individuals afflicted with periodontal disease, is significantly elevated compared to those with healthy periodontium, and the PGE2 levels in GCF serve as a critical biomarker for anticipating the progression of periodontitis [3]. Thromboxane A2 (TXA2) serves as a facilitator of vasoconstriction and an enhancer of platelet aggregation. As it is very unstable, therefore, it is present in the form of TXB2, which is present in abundance in the GCF [4,5]. TXB2 is found to increase in periodontitis and gingival inflammation [5]. Given their antagonistic roles, a meticulous equilibrium between PGE2 and TXB2 is imperative.

Type 2 diabetes mellitus (DM) is a metabolic disorder characterized by persistent hyperglycemia and diminished insulin secretion, leading to disruption in the transfer of glucose from the bloodstream into tissues, which subsequently results in elevated blood glucose concentrations. The alteration in the host's immunoinflammatory response to pathogens is pivotal in this context. DM can adversely affect the adhesion, chemotaxis, and phagocytic capacity of neutrophils, thereby facilitating bacterial survival within the periodontal pocket and significantly exacerbating periodontal tissue destruction. There exists substantial evidence indicating that the dysregulation of prostaglandins contributes to the protraction of wound healing processes [6]. In a study by Shaheen et al. [7], it was concluded that PGE2 levels were significantly increased in diabetic patients, compared to non-diabetic patients. However, no study has been conducted to assess the levels of TXB2 in diabetic patients with chronic periodontitis. Therefore, the primary objective of the present study was to assess the levels of TXB2 and PGE2 in the GCF of diabetic and non-diabetic patients with chronic periodontitis. The secondary objectives were to correlate these levels with various parameters such as age, gender, glycated hemoglobin (HbA1c) levels, PD, and fasting blood sugar levels and to determine the significant predictors for PGE2 and TXB2 levels. The null hypothesis posited for this study was that there would not be any difference in levels of TBX2 and PGE2 in diabetic and non-diabetic patients.

## Materials and methods

Study design

This cross-sectional, case-control study was conducted at the Department of Periodontology of Kothiwal Dental College and Research Centre, Moradabad, India, from July 2022 to August 2023. Approval was obtained from the institution's Institutional Ethics and Review Board (approval number: KDCRC/IERB/12/2022/76) before starting the study, and the study was conducted in accordance with the principles of the Declaration of Helsinki and Strengthening the Reporting of Observational Studies in Epidemiology (STROBE) guidelines. Written informed consent was obtained from all the patients.

Study population

The target population included 40 persons (cases) suffering from chronic generalized periodontitis with and without diabetes and 20 healthy patients (controls) without any systemic or localized infection. The cases were selected based on the following inclusion criteria: clinically and radiographically confirmed cases of chronic periodontitis [8] ≥18 years of age, who visited the department during the study period with the absence of any systemic problem except medically diagnosed type 2 DM with HbA1c levels tested in last six months, a minimum of ≥4 mm PD [9], a minimum of 15 teeth, and no periodontal therapy or antibiotic consumption in the past six months. The inclusion criteria for the control group were as follows: systemically and periodontally healthy individuals [8]. Pregnant and lactating females, individuals with multiple missing or decayed teeth with root stumps, individuals who consumed anti-inflammatory drugs such as non-steroidal anti-inflammatory drugs (NSAIDs) in the past six months, individuals with inflammatory disorders of the oral cavity, tobacco (smoke and smokeless) and alcohol users, and edentulous patients were excluded from the study.

Sample size estimation

The determination of the sample size was performed utilizing the effect size (Cohen's d) of 0.49, which was extrapolated from a prior investigation indicating a mean difference in PGE2 levels between the cohorts of 389.17 and a pooled standard deviation (SD) of 793.8. In order to attain a statistical power of 90% (β=0.10) alongside a significance threshold (α error) of 5%, a two-tailed statistical analysis was implemented [10]. By employing these parameters, a comprehensive sample size of 60 participants (20 individuals per group) was deemed adequate to identify a statistically significant disparity between the groups.

Group allocation

Sixty patients were divided equally into three groups: group 1 (n=20) comprising type 2 DM patients with chronic periodontitis, group 2 (n=20) comprising non-diabetic patients with chronic periodontitis, and group 3 (n=20) as controls comprising periodontally and systemically healthy individuals.

Methodology

All the patients were asked to self-check their fasting blood sugar levels on the day of the examination. HbA1c levels were recorded for all the patients on the same day. The periodontal condition of each participant was assessed utilizing the Periodontitis Index as established by Albandar [11], wherein individuals were categorized as experiencing mild, moderate, or severe periodontitis or as exhibiting no signs of periodontitis, predicated on the quantity (or proportions) of dentition displaying specific thresholds of PD and clinical attachment loss (CAL). The PD was evaluated at six designated sites per tooth. The PD and CAL were quantified utilizing a sterile calibrated probe (UNC-15, Hu-Friedy, Chicago, Illinois, United States). The bone loss was determined on both mesial and distal surfaces of all teeth by employing digital intraoral radiographs. All the clinical and radiographical investigations were carried out by a single-blinded, calibrated, and trained investigator (kappa scores of 0.92 and 0.94, respectively).

The GCF was collected using the following protocol: after achieving complete isolation using dry cotton rolls, and saliva ejectors, the tooth surfaces were meticulously dried. Three paper points (no. 25) were inserted into the periodontal pockets and maintained in position for a duration of 20-30 seconds. If the paper points were contaminated with blood, they were discarded. Subsequently, the non-contaminated paper points were transferred into sterile Eppendorf tubes (Eppendorf AG, Hamburg, Germany) containing 200 μl of phosphate-buffered saline, then refrigerated, and promptly conveyed to the biochemistry laboratory for further analysis. The Eppendorf tubes containing the samples were preserved at −70°C till further analysis. PGE2 levels were checked using an enzyme-linked immunosorbent assay (ELISA) kit (Biosource, Invitrogen Corporation, Carlsbad, California, United States). TXB2 was also tested using an ELISA kit (Abcam, Cambridge, United Kingdom). The kit was used following the manufacturer's instructions.

Statistical analysis

The data collected were entered into Microsoft Excel (Microsoft Corporation, Redmond, Washington, United States) and analyzed using IBM SPSS Statistics for Windows, Version 22.0 (Released 2013; IBM Corp., Armonk, New York, United States). The normality of data distribution was verified using the Kolmogorov-Smirnov test, and continuous variables were expressed as means and SD. Group comparisons were performed using a two-way analysis of variance (ANOVA) to evaluate the interaction effects between the different groups and factors. For assessing correlations between variables, Pearson correlation analysis was applied. Furthermore, principal component analysis (PCA) was conducted to reduce the dimensionality of the correlation matrix, allowing for the identification of underlying patterns among the variables while minimizing multicollinearity. A p-value of ≤0.05 was considered statistically significant throughout the analysis.

## Results

The study evaluated various clinical parameters across three groups. The mean age of the patients was highest in group 1 (49.1±6.37 years), followed by group 2 (43.45±8.18 years) and group 3 (43.8±8.37 years). Group 1 had a duration of diabetes, with a mean of 5.8±2.89 years. HbA1c levels were significantly higher in group 1 (9.79±1.22%), compared to group 2 (3.22±0.81%) and group 3 (3.25±0.73%). Fasting blood sugar levels were also elevated in group 1 (219.3±33.03 mg/dl), while lower in group 2 (88.25±9.65 mg/dl) and least in group 3 (85.95±9.3 mg/dl). PD was highest in group 1 (4.56±0.68 mm), followed by group 2 (4.28±0.81 mm), and lowest in group 3 (1.7±0.22 mm). PGE2 and TXB2 levels were notably elevated in group 1 and least in group 3 (Table 1).

**Table 1:** Descriptive analysis of the study sample. SD: standard deviation; HbA1c: glycated hemoglobin; PGE2: prostaglandin E2; TXB2: thromboxane B2

Parameters	Groups	Frequency	95% confidence interval for mean	Mean±SD
Age (years)	Group 1	20	46.12-52.08	49.1±6.37
	Group 2	20	39.62-47.28	43.45±8.18
	Group 3	20	39.88-47.72	43.8±8.37
Duration of diabetes (years)	Group 1	20	4.45-7.15	5.8±2.89
	Group 2	20	0-0	0±0
	Group 3	20	0-0	0±0
HbA1c (%)	Group 1	20	9.22-10.36	9.79±1.22
	Group 2	20	2.84-3.59	3.22±0.81
	Group 3	20	2.91-3.59	3.25±0.73
Fasting blood sugar (mg/dl)	Group 1	20	203.84-234.76	219.3±33.03
	Group 2	20	83.74-92.76	88.25±9.65
	Group 3	20	81.6-90.3	85.95±9.3
Probing depth (mm)	Group 1	20	4.25-4.88	4.56±0.68
	Group 2	20	3.9-4.65	4.28±0.81
	Group 3	20	1.59-1.81	1.7±0.22
PGE2 (pg/mL)	Group 1	20	3623.48-4086.52	3855±494.68
	Group 2	20	1867.15-2459.85	2163.5±633.22
	Group 3	20	197.78-265.22	231.5±72.06
TXB2 (pg/mL)	Group 1	20	1410.38-1670.82	1540.6±278.24
	Group 2	20	795.12-956.88	876±172.82
	Group 3	20	115.87-151.53	133.7±38.09

As statistically significant differences were observed between the groups, the null hypothesis was rejected in our study. The two-way ANOVA test results revealed statistically significant differences across groups for all parameters. PGE2 levels showed a highly significant difference (p=0.001) with a large effect size (η²=0.91). Similarly, TXB2 levels demonstrated significant variation between groups (p=0.001, η²=0.91). PD also had a significant difference (p=0.001, η²=0.82). HbA1c (p=0.001, η²=0.92) and fasting blood sugar levels (p=0.001, η²=0.91) showed highly significant differences as well. Age exhibited a significant but smaller effect size (p=0.041, η²=0.11). These results indicated strong group differences, particularly in biochemical parameters (Table 2).

**Table 2:** Comparison of groups with respect to various parameters using ANOVA test. df: degree of freedom; *p-value ≤0.05: significant; HbA1c: glycated hemoglobin; PGE2: prostaglandin E2; TXB2: thromboxane B2; ANOVA: analysis of variance

Parameters	Sum of squares	df	Mean square	F value	P-value	η²
PGE2 (pg/mL)	131490323.33	2	65745161.67	303.03	0.001*	0.91
TXB2 (pg/mL)	19813800.4	2	9906900.2	273.32	0.001*	0.91
Probing depth (mm)	99.49	2	49.74	128.28	0.001*	0.82
HbA1c (%)	573.36	2	286.68	322.34	0.001*	0.92
Fasting blood sugar (mg/dl)	233077.43	2	116538.72	275.21	0.001*	0.91
Age (years)	400.9	2	200.45	3.39	0.041*	0.11

Pearson correlation analysis revealed significant relationships between various parameters. HbA1c levels showed a strong positive correlation with fasting blood sugar levels (r=0.91, p=0.001), duration of diabetes (r=0.85, p=0.001), and PGE2 levels (r=0.82, p=0.001). Fasting blood sugar levels were also strongly correlated with the duration of diabetes (r=0.79, p=0.001), PGE2 (r=0.83, p=0.001), and TXB2 (r=0.91, p=0.001). PD had a moderate correlation with HbA1c (r=0.51, p=0.001), fasting blood sugar levels (r=0.51, p=0.001), and PGE2 (r=0.83, p=0.001). Age showed weaker but still significant correlations with HbA1c (r=0.32, p=0.013) and fasting blood sugar (r=0.32, p=0.014), while TXB2 was highly correlated with both PGE2 (r=0.91, p=0.001) and other metabolic indicators, confirming significant interrelationships between diabetes and periodontal health parameters (Table 3).

**Table 3:** Correlation between various parameters using Pearson correlation test. *p-value ≤0.05: significant; HbA1c: glycated hemoglobin; PGE2: prostaglandin E2; TXB2: thromboxane B2 Very weak correlation: 0.0<∣r∣<0.20; weak correlation: 0.2≤∣r∣<0.4; moderate correlation: 0.4≤∣r∣<0.6; strong correlation: 0.6≤∣r∣<0.8; very strong correlation: 0.8≤∣r∣≤1

Parameters	Correlation with significance	HbA1c	Fasting blood sugar	Duration of diabetes	Age	Probing depth	PGE2
HbA1c (%)	Correlation	1					
	P-value						
Fasting blood sugar (mg/dl)	Correlation	0.91	1				
	P-value	0.001*					
Duration of diabetes	Correlation	0.85	0.79	1			
	P-value	0.001*	0.001*				
Age (years)	Correlation	0.32	0.32	0.43	1		
	P-value	0.013*	0.014*	0.001*			
Probing depth (mm)	Correlation	0.51	0.51	0.55	0.28	1	
	P-value	0.001*	0.001*	0.001*	0.029*		
PGE2 (pg/mL)	Correlation	0.82	0.83	0.81	0.35	0.83	1
	P-value	0.001*	0.001*	0.001*	0.007*	0.001*	
TXB2 (mg/mL)	Correlation	0.88	0.91	0.76	0.24	0.71	0.91
	P-value	0.001*	0.001*	0.001*	0.065	0.001*	0.001*

The linear regression analysis revealed several significant predictors for PGE2 and TXB2 levels. For PGE2, fasting blood sugar levels (p=0.006) and PD (p<0.001) were significant predictors, while HbA1c (p=0.284), duration of diabetes (p=0.735), and age (p=0.635) were not significant. For TXB2, significant predictors included HbA1c (p=0.003), fasting blood sugar levels (p=0.015), and PD (p<0.001). Age (p=0.234) and duration of diabetes (p=0.078) were not significant predictors for TXB2. Overall, periodontal PD and fasting blood sugar levels showed the strongest influence on both PGE2 and TXB2 levels (Table 4).

**Table 4:** Linear regression analysis for PGE2 and TXB2 levels. *p-value ≤0.05: significant; HbA1c: glycated hemoglobin; PGE2: prostaglandin E2; TXB2: thromboxane B2

Parameters	PGE2		TXB2	
Model	Coefficients	P-value	Coefficients	P-value
(Constant)	-1321.91	0.019*	-443.76	0.045*
HbA1c (%)	72.35	0.284	82.26	0.003*
Fasting blood sugar (mg/dl)	8.09	0.006*	2.83	0.015*
Duration of diabetes (years)	16.99	0.735	-35.68	0.078
Age (years)	-5.15	0.635	-5.17	0.234
Probing depth (mm)	612.97	0.001*	222.25	0.001*

**Figure 1:**
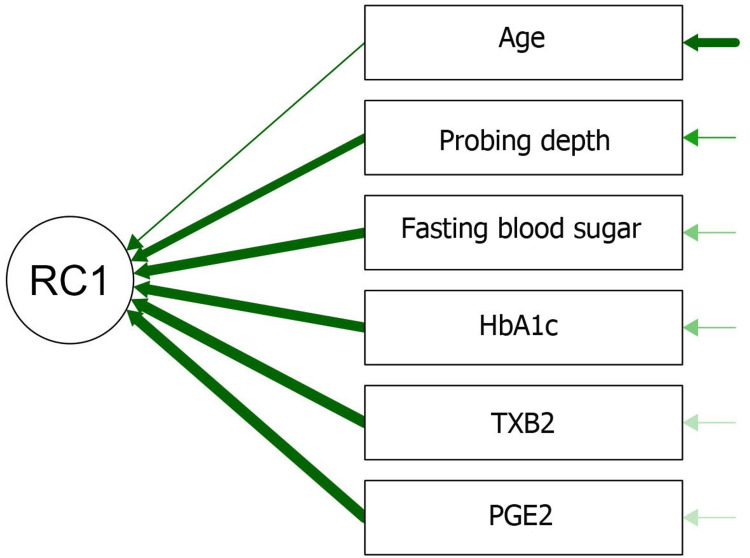
Path diagram for principal components. HbA1c: glycated hemoglobin; PGE2: prostaglandin E2; TXB2: thromboxane B2

The results of the PCA indicated that the first principal component (RC1) is strongly influenced by several variables. Both HbA1c and fasting blood sugar levels exhibited high loadings of 0.89, suggesting that these two variables contribute significantly to the variation captured by RC1. Additionally, the markers PGE2 and TXB2 display very high loadings of 0.95 and 0.94, respectively, indicating that they are the dominant contributors to RC1. This suggested that the inflammatory markers PGE2 and TXB2 are particularly important in explaining the variation in this dataset. The PD also played a notable role, with a loading of 0.8, showing that it has a strong relationship with the RC1. In contrast, age has a lower loading value of 0.41, indicating that it contributed less to RC1 compared to the other variables. Overall, the results suggested that RC1 is primarily driven by metabolic (blood sugar levels) and inflammatory (PGE2 and TXB2) factors, with some contribution from periodontal conditions and age. This component likely reflects a combination of systemic and local health factors in the patient population (Figure 1).

**Figure 2:**
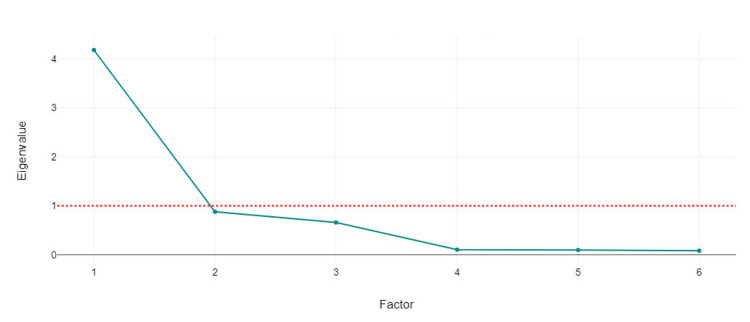
Eigenvalues for six components in PCA. PCA: principal component analysis

The eigenvalues of each principal component are plotted against their respective factors (Figure 2).

This type of plot is commonly known as a scree plot. All six factors are shown in Figure 1. The plot vividly showed a steep decline from the first to the second factor, indicating that the first factor captured a significant portion of the variance within the dataset. After the second factor, the curve flattened considerably, suggesting that additional factors contributed progressively less to explaining the variance. According to the Kaiser criterion, only the factors with eigenvalues above the red dotted line are considered significant. In this case, the plot suggested that the first two factors are significant as they were above the cut-off line, with the subsequent factors dropping below it and thus likely offering diminishing returns on information about the data structure (Figure 2).

## Discussion

Inflammatory mediators present in the GCF have been identified as potential biomarkers for both the advancement and intensity of periodontitis, in addition to serving as indicators for therapeutic response. In the current investigation, the average concentrations of PGE2 and TBX2 in the GCF were observed to be significantly lower in the control group compared to those with chronic periodontitis. This was in accordance with previous studies [3,5,12].

Prostaglandins are synthesized through a sequential process involving three enzymatic reactions: the liberation of arachidonic acid (AA) from phospholipid membrane constituents via phospholipases, the transformation of AA into the unstable endoperoxide intermediate, prostaglandin H2 (PGH2), mediated by cyclooxygenase (COX), and the isomerization of PGH2 into bioactive prostanoids such as PGE2, prostacyclin, prostaglandin F2, prostaglandin D2, and TXA2, facilitated by specific terminal synthases. TXA2, being an unstable biomarker, gets converted into TXB2 [4,5]. Their levels increased in cases of chronic inflammation, which might be a reason for increased levels of PGE2 and TXB2 in our cases compared to controls with healthy periodontium.

When, among the cases, the levels of biomarkers were compared between diabetic and non-diabetic patients, the results of our study revealed that the levels of biomarkers were more in diabetic patients, compared to non-diabetic patients. This observation could not be juxtaposed with existing literature concerning TXB2, as no investigations have been conducted on this subject matter. Conversely, regarding PGE2, our findings align with those of previous research [7,10,13]. This indicates that diabetes, as a chronic inflammatory condition, results in elevated concentrations of inflammatory cytokines such as PGE2 within the circulatory system.

The results of the study further revealed that the levels of both biomarkers showed a positive correlation with HbA1c, PD, fasting blood sugar levels, and duration of type 2 DM. Furthermore, PD and fasting blood sugar levels showed the strongest influence on both PGE2 and TXB2 levels. These empirical findings act as a reconfirmation of the well-established scientific understanding that a chronic condition of hyperglycemia, commonly observed in individuals with inadequately managed DM, intensifies inflammatory responses via the increased production of advanced glycation end products (AGEs) in GCF [6]. Similar results were reported in previous studies [7,10]. It is thus hypothesized that a condition of chronic hyperglycemia in individuals belonging to group 1 has precipitated a state of oxidative stress within the periodontal tissues of these patients, consequently aggravating both the clinical and radiographic indicators of periodontal inflammation.

Our study revealed that PD was significantly increased in group 1, compared to other groups, with a concomitant increase in the levels of biomarkers. This finding was in contradiction with the findings of Shaheen et al. [7], who found similar periodontal status in groups 1 and 2. This could have been due to the fact their study included patients with controlled type 2 DM (HbA1c levels of 5%), whereas, in our study, the patients in group 1 had uncontrolled type 2 DM (HbA1c levels of 9%). This indicated that in cases of poorly regulated type 2 DM and prolonged diabetes duration, heightened periodontal damage is observed alongside elevated concentrations of inflammatory biomarkers, including PGE2 and TXB2 [6].

The findings of our study revealed a weak correlation between age and levels of inflammatory biomarkers. This could be because the inflammatory responses within the periodontium are predominantly influenced by periodontal pathology. The degree or advancement of periodontal disease, which does not necessarily align with chronological age, may play a more significant role in ascertaining the levels of inflammatory biomarkers. For example, an individual of a younger age suffering from severe periodontitis could exhibit elevated inflammatory markers compared to an older individual with a milder form of the disease [14].

Clinical implications of the study

Our research findings serve to further validate and reinforce the conclusions drawn from previous studies, which fundamentally highlight the indispensable role that effective glycemic control plays in the maintenance of a stable and consistent periodontal health status among individuals who have been clinically diagnosed with DM. It is of paramount importance that community-based health education initiatives be systematically and routinely implemented to effectively disseminate knowledge and raise awareness among the public regarding the intricate relationship that exists between systemic conditions such as DM and various oral inflammatory diseases, including but not limited to periodontal diseases. Furthermore, these educational initiatives ought to place significant emphasis on elucidating the detrimental effects that can arise from persistently elevated blood glucose levels, commonly referred to as hyperglycemia, while also articulating the substantial benefits associated with maintaining optimal glycemic control, particularly concerning not only periodontal health but also the broader spectrum of overall well-being and health outcomes. In this regard, the educational content provided in these programs must be both comprehensive and accessible, thus ensuring that individuals are fully informed about the critical interconnectedness of systemic health and oral health, ultimately fostering a more health-conscious society.

Limitations of the study

Our study did not evaluate the subgingival flora in the study groups, which would provide more information on the relationship between DM and periodontitis. Furthermore, the current study did not assess sex differences in the levels of inflammatory biomarkers. Our study has excluded individuals using nicotine products, which could have a synergistic effect with DM. Therefore, future studies are required in this direction.

## Conclusions

The findings of the study revealed that hyperglycemia associated with type 2 DM led to a significant increase in the levels of PGE2 and TXB2 in the GCF of patients with chronic periodontitis. The levels of both biomarkers showed a positive correlation with HbA1c, PD, fasting blood sugar levels, and duration of type 2 DM. For PGE2, fasting blood sugar levels and PD were significant predictors, whereas, for TXB2, significant predictors included HbA1c, fasting blood sugar levels, and PD. Age showed a weak positive correlation with both biomarkers.
